# Multilevel Cortical Processing of Somatosensory Novelty: A Magnetoencephalography Study

**DOI:** 10.3389/fnhum.2016.00259

**Published:** 2016-06-02

**Authors:** Gilles Naeije, Thibaut Vaulet, Vincent Wens, Brice Marty, Serge Goldman, Xavier De Tiège

**Affiliations:** Laboratoire de Cartographie fonctionnelle du Cerveau (LCFC), UNI–ULB Neuroscience Institute, and Magnetoencephalography Unit, ULB-Hôpital Erasme, Université libre de Bruxelles (ULB)Brussels, Belgium

**Keywords:** predictive coding, change detection, somatosensory, mismatch negativity, P300, contingent magnetic variation

## Abstract

Using magnetoencephalography (MEG), this study investigates the spatio-temporal dynamics of the multilevel cortical processing of somatosensory change detection. Neuromagnetic signals of 16 healthy adult subjects (7 females and 9 males, mean age 29 ± 3 years) were recorded using whole-scalp-covering MEG while they underwent an oddball paradigm based on simple standard (right index fingertip tactile stimulation) and deviant (simultaneous right index fingertip and middle phalanx tactile stimulation) stimuli gathered into sequences to create and then deviate from stimulus patterns at multiple (local vs. global) levels of complexity. Five healthy adult subjects (3 females and 2 males, mean age 31, 6 ± 2 years) also underwent a similar oddball paradigm in which standard and deviant stimuli were flipped. Local deviations led to a somatosensory mismatch response peaking at 55–130 ms post-stimulus onset with a cortical generator located at the contralateral secondary somatosensory (cSII) cortex. The mismatch response was independent of the deviant stimuli physical characteristics. Global deviants led to a P300 response with cortical sources located bilaterally at temporo-parietal junction (TPJ) and supplementary motor area (SMA). The posterior parietal cortex (PPC) and the SMA were found to generate a contingent magnetic variation (CMV) attributed to top-down expectations. Amplitude of mismatch responses were modulated by top-down expectations and correlated with both the magnitude of the CMV and the P300 amplitude at the right TPJ. These results provide novel empirical evidence for a unified sensory novelty detection system in the human brain by linking detection of salient sensory stimuli in personal and extra-personal spaces to a common framework of multilevel cortical processing.

## Introduction

The ability to evolve safely in an environment highly depends on the ability to detect novel and unexpected events. This aptitude relies on preattentive neural processes that efficiently discriminate novel behaviorally relevant sensory inputs from irrelevant stimuli and attract attention towards these salient sensory stimuli to allow conscious awareness and appropriate behavioral response reaction.

According to the Bayesian brain and predictive coding theories, the human brain generates an internal model of the world that predicts sensory inputs based upon prior experiences (for a review, see Friston, 2010). When top-down predictions and bottom-up sensory inputs do not match, the prediction error is conveyed from low-level sensory cortices to high-level associative cortices to optimize top-down predictions (Friston, 2010). High-level cortical areas then pass updated predictions via feedback projections to lower levels to minimize the prediction error (Friston, 2010). The prediction error associated with a given sensory input or its “surprise” is the information ultimately taken into account for behavioral processing (Friston, 2010).

Classical electrophysiological markers of top-down prediction violation by bottom-up sensory inputs are novelty-evoked responses such as the mismatch negativity (MMN; for a review, see Garrido et al., 2009b) and the P300 (Polich, 2007; Garrido et al., 2009a; Ostwald et al., 2012). The MMN is considered to be a preattentive cortical response typically elicited by a deviant stimulus embedded in a sequence of identical standard stimuli (Garrido et al., 2009b). Within the predictive coding framework, the MMN would reflect the prediction error signal generated by lower-level sensory areas after top-down priors violation occurring over a short timescale (Garrido et al., 2009b; Friston, 2010). By contrast, the P300, which depends on attention, is thought to reflect violation of expected complex patterns of sensory stimulations occurring over longer timeframes in high-level associative cortical areas (Bekinschtein et al., 2009). This late cortical response is regarded as an index of working memory update (Polich, 2007). Finally, several studies have reported a slow baseline drift that builds up in the absence of prediction errors, coined the contingent negative variation (CNV) in electroencephalography (EEG; Faugeras et al., 2012; Chennu et al., 2013). The CNV is considered to reflect progressive changes in expectation based on the hypothesis that expectation about incoming stimuli is supposed to strengthen with each correct prediction of sensory inputs (Chennu et al., 2013).

Interestingly, the integration of those different electrophysiological responses within the context of predictive coding has been achieved for the auditory modality. This was accomplished by using an oddball design based on simple tones gathered into sequences to create stimulus patterns at multiple levels of predictive complexity and then deviate from them (Bekinschtein et al., 2009; Wacongne et al., 2011; Chennu et al., 2013). In addition, experimental evidence supporting a hierarchical processing of sensory stimuli has been obtained for the visual modality (Rao and Ballard, 1999; Foxe et al., 2002 ). Taken together, these data support the existence of a common framework for the detection of novel incoming sensory stimuli occurring in the extrapersonal space. By contrast, evidence supporting similar multilevel cortical processing for the somatosensory modality, which typically reflects personal space sensory processing, are scarce. Still, P300 responses obtained using somatosensory oddball paradigms have been associated with conscious change detection (Downar et al., 2000; Hashimoto et al., 2000; Huang et al., 2005; Kida et al., 2012; Lugo et al., 2014). Furthermore, previous somatosensory MMN (sMMN) studies have mostly focused on the temporal dynamics of this mismatch response, while those investigating the sMMN spatial dynamics led to rather conflicting results (Kekoni et al., 1997; Shinozaki et al., 1998; Akatsuka et al., 2005; Restuccia et al., 2007; Spackman et al., 2007; Butler et al., 2011; Strömmer et al., 2014). In addition, to the best of our knowledge, only one study provided some empirical evidence supporting the involvement of the predictive coding mechanism in sMMN by showing that this response reflects unpredicted somatosensory stimuli rather than stimulus change *per se* (Ostwald et al., 2012). Finally, CNV investigations with oddball paradigms have been limited to extrapersonal sensory stimuli, either auditory (Faugeras et al., 2012; Chennu et al., 2013) or visual (Mento, 2013), and its cortical generators remain poorly understood (Elbert et al., 1994; Babiloni et al., 2005).

Here, we adapted the integrative oddball paradigm developed for the auditory modality (Bekinschtein et al., 2009; Wacongne et al., 2011; Chennu et al., 2013) to the somatosensory (tactile) modality. This oddball paradigm was initially designed to simultaneously investigate the multiple levels of cortical processing involved in auditory change detection at both the preattentional and the attentional levels. This magnetoencephalography (MEG) study therefore aimed at characterizing the spatio-temporal dynamics of the multilevel cortical processing involved in somatosensory change detection by studying the magnetic counterparts of the sMMN (magnetic sMMN (msMMN)), the P300 and the CNV (contingent magnetic variation (CMV)) in the sensor and the source spaces. If somatosensory change detection involves multilevel cortical processing patterns similar to those in auditory and visual change detection, this study would provide novel empirical data supporting a unified account for the cortical processing of novel sensory stimuli in the human brain.

## Materials and Methods

### Subjects

Sixteen healthy adult subjects (mean age 29 ± 3 years, 7 females and 9 males) without any history of neurological or psychiatric disorder were studied.

All subjects were right-handed according to the Edinburgh handedness inventory (Oldfield, 1971). They participated after written inform consent. The study was approved by the ULB-Hôpital Erasme Ethics Committee (Reference EudraCT/CCB : B406201317212).

### Experimental Paradigm

**Figure 1:**
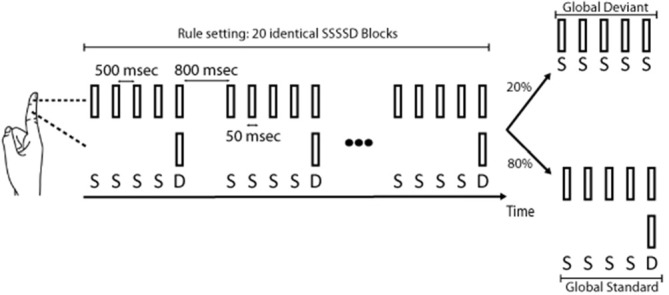
**Somatosensory oddball paradigm used in this study.** In sessions 1 and 2a, standards (S) corresponded to a pneumatic tactile stimulation of the right index fingertip, and deviants (D) to a similar tactile stimulation but simultaneously applied to the fingertip and the middle phalanx of the right index. Blocks of five stimuli either comprised four standards followed by a deviant (four standard stimuli followed by a deviant stimulus (standard (SSSSD) blocks) or five standards (deviant SSSSS blocks). Each deviant in SSSSD blocks (local deviation), by breaking a sequence of four identical stimuli, elicited a magnetic somatosensory MMN (msMMN) response. SSSSS blocks, by breaking the learned rule, evoked a P300 response. In session 2b, a similar stimulation paradigm was used except that standards and deviants were flipped compared to sessions 1 and 2a. In session 2c, standard blocks and deviant blocks were flipped compared to sessions 1 and 2a.

Figure 1 illustrates the somatosensory oddball paradigm used in this study.

During MEG recordings, subjects sat comfortably in the MEG chair while they underwent a unilateral somatosensory oddball paradigm adapted from the auditory local/global oddball paradigm of Bekinschtein et al. (2009) and Chennu et al. (2013). In this oddball experiment (session 1), standard stimuli (standards, S) corresponded to a pneumatic tactile stimulation (stimulated area: 1 cm^2^, intensity: 2 bars, duration: 50 ms) applied to the right index fingertip. Deviant stimuli (deviants, D) were similar tactile stimulations but simultaneously applied to the fingertip and the middle phalanx of the right index. Blocks of five stimuli were applied with an inter-stimulus interval (ISI) of 500 ms and either comprised four standard stimuli followed by a deviant stimulus (SSSSD) or five standard stimuli (SSSSS). One hundred and twenty blocks (inter-block interval (IBI): 800 ms) were administered to the subjects. The first 20 blocks were always SSSSD blocks (standard blocks) and in the subsequent 100 blocks, 20 SSSSS blocks (deviant blocks) were randomly intermingled among SSSSD blocks (with the only constraint that two SSSSS blocks could not occur successively). Subjects were informed at the beginning of the experiment that the first 20 blocks (SSSSD) corresponded to the pattern they had to remember as the rule. They were also asked to count the number of SSSSS blocks that broke the learned rule. Each deviant stimulus within the 100 SSSSD blocks, by breaking a sequence of four identical stimuli, led to a local deviation (local deviants) that was expected to generate a msMMN (Bekinschtein et al., 2009; Wacongne et al., 2011; Chennu et al., 2013). Furthermore, if msMMN responses are indeed modulated by top-down expectation, local deviants in standard blocks (SSSSD blocks) occurring immediately after deviant blocks (SSSSS blocks) should lead to higher msMMN responses than those occurring before since the deviant stimulus should be less expected in the former case. Each deviant SSSSS block intermingled with the standard SSSSD blocks led to a global deviation (global deviants) expected to evoke the P300 response. Only 20 deviant SSSSS blocks were used to ensure a low predictability of occurrence and to limit attentional fluctuations due to an excessive duration of the task. Furthermore, previous auditory studies have demonstrated that 20 trials per subject were sufficient to obtain a reliable P300 response at the group level (Bekinschtein et al., 2009; Wacongne et al., 2011; Chennu et al., 2013). Subjects were asked to count the number of deviant SSSSS blocks to assess their understanding of the task and to maintain their attention during the experiment.

These local and global deviances were also used to derive the CMV (Faugeras et al., 2012; Chennu et al., 2013) characterized by a baseline drift that was expected to occur both at the intra- and the inter-block levels. At the intra-block level, the CMV was expected to build up within standard SSSSD blocks through the repetition of the four standard stimuli until the expected local deviant. At the inter-block level, a progressive baseline drift was supposed to appear after each successive presentation of standard SSSSD blocks until rule breaking by a deviant SSSSS block.

To confirm that msMMN responses elicited by this oddball paradigm were not due to differences in the physical characteristics of the deviant stimulus (i.e., for standards, the right index fingertip was stimulated (stimulated area: 1 cm^2^) whereas for deviants, the fingertip and the middle phalanx of the right index were stimulated (stimulated area: 2 cm^2^)), five subjects (mean age 31.6 ± 2 years, 3 females and 2 males) from the initial group of 16 subjects underwent three additional experiments in a separate session (session 2): (2a) the oddball paradigm described above; (2b) a similar oddball paradigm in which standard and deviant stimuli were flipped (i.e., standards becoming tactile stimulation simultaneously applied to the fingertip and the middle phalanx of the right index and deviants corresponding to a tactile stimulation applied to the right index fingertip); and (2c) a similar oddball paradigm in which standard and deviant blocks were flipped (i.e., SSSSS blocks establishing the rule (standard blocks, 20 first blocks plus 80 subsequent occurrences) and SSSSD blocks breaking the rule (global deviants, 20 blocks randomly intermingled with the 80 SSSSS blocks); standard and local deviant stimuli being identical as in sessions 1 and 2a). Session 2b was used as a control condition to confirm that the msMMN responses observed in the initial oddball paradigm (sessions 1 and 2a) were not merely due to a change in the physical characteristics of the deviant stimuli (compared with standard stimuli) but were mainly driven by prediction error detection. Furthermore, if the msMMN amplitude was higher in session 2c—where local deviants are less predictable—than in session 2a, this would imply that expectation modulates the sMMN response, suggesting a neural mechanism relying upon prediction error detection.

During the whole experiment, subjects wore earplugs to suppress the auditory noise associated with the pneumatic stimulation.

### Data Acquisition

Somatosensory evoked magnetic fields (SEFs) were recorded using a whole-scalp-covering MEG (Vectorview, Elekta Oy, Helsinki, Finland) installed in a lightweight magnetic shielded room (Maxshield, Elekta Oy, Helsinki, Finland; De Tiège et al., 2008). The MEG sensor layout consisted in 102 sets, each comprising one magnetometer and two orthogonal planar gradiometers with different spatial sensitivity (i.e., lead field) to right beneath or nearby neural sources. Four head-tracking coils monitored subjects’ head position inside the MEG helmet. The location of the coils and at least 150 head-surface (on scalp, nose and face) points with respect to anatomical fiducials were determined with an electromagnetic tracker (Fastrak, Polhemus, Colchester, VT, USA). Eye movements and blinks were monitored with vertical and horizontal electrooculograms (EOGs). Electrocardiogam (ECG) was recorded using bipolar electrodes placed below the clavicles. All signals were bandpassed at 0.1–330 Hz and sampled at 1 kHz. Subjects’ high-resolution 3D-T1 cerebral magnetic resonance images (MRIs) were acquired on a 1.5 T MRI scanner (Intera, Philips, Netherlands).

### Data Preprocessing and Sensor-Space Analyses

Continuous MEG data obtained in session 1 and 2 were first preprocessed off-line with the signal space separation (SSS) method to subtract external interferences and correct for head movements (Taulu et al., 2005; number of harmonic components kept inside the sensors array: *L*_in_ = 80). For msMMN, P300 and CMV analyses performed at the group level (session 1), the SSS method was also used to align subjects’ head to a common sensor space centered on a mean head position. Then, ocular and cardiac artifacts were subtracted from filtered data (off-line band-pass filter: 0.1–45 Hz) using independent component analysis as implemented in the FastICA algorithm (dimension reduction to 30, nonlinearity *tanh*; Vigário, 1997). Artifactual components were identified using temporal correlations with EOG and ECG (correlation thresholds: 0.15) and visual inspection of their spatial topography (number of rejected components ranged from 2 to 5 across subjects).

The open source software Fieldtrip^1^ was then used for further preprocessing (Oostenveld et al., 2011). A common preprocessing stream was used to investigate the evoked responses of interest, and included MEG data epoching, automatic (thresholds for epoch rejection: 0.7 pT/cm for planar gradiometers and 3 pT for magnetometers) and visual artifact rejection, low-pass filtering, baseline correction and epoch averaging.

For the msMMN, the epoch length was 800 ms (−200 ms to +600 ms post-stimulation onset), the low-pass filter was set to 45 Hz and the baseline was adjusted using the 150 ms pre-stimulus interval. In session 1 and session 2 (a, b, c), epochs corresponding to standard stimuli and to local deviants of SSSSD blocks were separately averaged. Individual-level averaged epochs from session 1 were also averaged across subjects (grand average). Differences between: (i) the resulting sensor-space standard and deviant SEFs in session 1 (individual and group levels) and session 2 (a, b and c); (ii) the resulting sensor-space deviant SEFs in sessions 2a vs. 2b and sessions 2a vs. 2c; and (iii) the resulting sensor-space standard SEFs in session 2a, 2b and 2c, were then statistically investigated (planar gradiometers only) between 20 and 200 ms post-stimulus onset at the individual level using a non-parametric cluster-based two-sided *t*-test as described by Maris and Oostenveld (2007) and implemented in Fieldtrip. Briefly, *t*-values assessing the response differences at each sensor and time sample were computed as well as their uncorrected *p*-values (two-sided *t*-test). To address the multiple spatiotemporal comparisons (204 channels and 180 time samples), clusters of adjacent spatiotemporal points were obtained using a 0.05 threshold on those *p*-values (with the constraint that at least two neighbor sensors are involved). Each cluster was weighted by their summed *t*-values and the maximum weight over all clusters was taken as statistic. Its significance was then assessed non-parametrically with the Monte-Carlo approach, i.e., its *p*-value was derived from a null distribution estimated using the maximum cluster weights of 1000 simulated datasets obtained by random permutations of standard and deviant epochs for (i) and paradigm labels 2a, 2b, 2c for (ii and iii). This method has the advantage of not implying any spatiotemporal *a priori* and allows localizing, using the maximum cluster at *p* < 0.025 (two-sided test), where and when SEFs were significantly different (notwithstanding interpretation issues discussed in Maris and Oostenveld, 2007).

For the P300, the same epoch length and baseline adjustment were used but the low-pass filter was set to 20 Hz. In session 1, epochs corresponding to the first four standard stimuli and the fifth standard stimulus (eliciting the global deviation) of all SSSSS blocks were separately averaged. The resulting individual-level SEFs were subsequently averaged across subjects (grand average) to optimize the signal-to-noise ratio (low number of global deviants per subject). Sensor-space (planar gradiometers only) statistical differences between the averaged first four and the fifth standards of SSSSS blocks were then assessed at the group level between 250 and 600 ms post-stimulus using a non-parametric cluster-based approach similar to that described above, but with two differences: the time interval of interest comprised 350 time samples, and the two conditions (averaged first four S vs. fifth S) were randomly permuted subject-wise rather than epoch-wise.

For the CMV, the epoch length was set to 700 ms (−500 ms to +200 ms post-stimulation onset) for intra-block and 2400 ms (−2200 ms to +200 ms post-deviant onset in SSSSD blocks) for inter-block CMV drift analyses. The low-pass filter was set to 20 Hz. All epochs were baseline corrected using their first 100-ms. For intra-block CMV analyses, epochs corresponding to the first standard stimuli and the local deviants of all standard SSSSD blocks were separately averaged within and across (grand average) subjects. For inter-block CMV analyses, epochs corresponding to the whole time course of standard SSSSD blocks occurring before and after deviant SSSSS blocks were likewise separately averaged within and across (grand average) subjects. Slopes of the resulting grand averaged SEF time courses were estimated by linear regression, and their differences were then investigated between those of the first standard stimulus and the local deviant of standard SSSSD blocks (intra-block CMV), and between those of standard SSSSD blocks occurring before and after deviant SSSSS blocks (inter-block CMV). Statistically significant group differences between the individual-level slopes before and after rule-breaking, were identified using a permutation-based test (Nichols and Holmes, 2002). Under the null hypothesis of no mean difference across subjects, the slopes before and after rule-breaking were exchangeable. We thus generated a null distribution of 10^4^ mean slopes differences by applying this exchange on a random selection of subjects before group averaging (Nichols and Holmes, 2002). This was performed independently for each of the 306 sensors. To control the family-wise error associated with those multiple comparisons, we used a Bonferroni correction for the number of degrees of freedom present in the MEG data, which can be estimated as the SSS parameter *L*_in_ = 80, leading to an effective significance level of *p* < 0.05/*L*_in_ = 0.0006. Any sensor whose mean slope difference presented a *p*-value (derived from the null distribution) lower than this value was deemed significant.

### Source Reconstruction and Source-Space Analyses

Neural generators of the msMMN, the P300 and the CMV (session 1 only for the P300 and the CMV) were identified using conventional equivalent current dipole (ECD) modeling tools (Elekta Oy, Helsinki, Finland) using an approach described in details in Salmelin (2010). ECD modeling was chosen over distributed or beamformer methods because the available literature suggests that the number of cortical generators for msMMN, P300 and CMV is limited (Dammers and Ioannides, 2000; Downar et al., 2000; Gómez et al., 2004; Strömmer et al., 2014). A spherical conductor model determined from the subjects’ MRI (individual-level analyses; msMMN) or the Montreal Neurological Institute (MNI) template (group-level analyses; msMMN, P300, CMV) was used. For msMMN and P300, magnetic field patterns were visually inspected during the timeframe identified by the sensor-level cluster-based statistical approach described above (see “Data Preprocessing and Sensor Space Analysis” Section). Clear dipolar field patterns were used in a nonlinear search to localize the corresponding source. When appropriate, to optimize ECD spatial accuracy and avoid any influence of irrelevant magnetic signals, dipole fitting was performed using a selection of at least 40 sensors centered over the maximal magnetic fields difference between standards and deviants of standard SSSSD blocks for the msMMN, and between the first four standard stimuli and the fifth standard stimulus of all deviant SSSSS blocks for the P300 (see “Data Preprocessing and Sensor Space Analysis” Section). For the CMV, ECD(s) that best explained the baseline drift were obtained using a selection of at least 40 sensors centered over the maximal slope difference (see “Data Preprocessing and Sensor Space Analysis” Section).

Only dipoles with a goodness of fit above 85% were considered as relevant and their source strength waveforms were then estimated over the considered epochs. When multiple sources were required to explain the magnetic field patterns, the number of ECDs was determined based on classical multidipole modeling approaches that relied on: (1) the number of clear dipolar magnetic field patterns; (2) the correspondence between the original data and the predicted source activity at the sensor level of each source or their combination; (3) the use of source activity linear projection from original sensor level data; and (4) the goodness of fit of the multidipole modeling (Salmelin, 2010). Finally, the source strength waveforms of each ECD over the whole epoch were obtained from the single or multi-dipole model. ECDs were superimposed on the co-registered subjects’ MRI (individual-level analyses; msMMN) or MNI template (group-level analyses; msMMN, P300, CMV).

Finally, to further confirm the sensor-space tests for differences between standards and deviants, *post hoc* paired *t*-tests were applied on the corresponding sources intensity (standards or deviants) at the timing of maximum source amplitude within the time frame disclosed by sensor-level analyses. Then, to search for an effect of top-down predictions on msMMN amplitude, paired *t*-tests were also applied at the individual level on sources intensity of local deviants within the standard SSSSD blocks occurring before and after deviant SSSSS blocks in session 1, and on the corresponding sources intensity of deviants in sessions 2a vs. 2c.

### Correlation Analyses

Spearman’s rank correlation test was used to investigate possible interactions between the msMMN, the P300 and the inter-block CMV responses observed in session 1. More specifically, this non-parametric dependency test was used to assess the existence of monotonic relationships across participants between these neural responses. In practice, we searched for significant inter-subject correlation between: (1) individual source-level msMMN amplitude and P300 amplitude; (2) the individual difference in source-level msMMN amplitude and the strength of inter-block CMV (i.e., maximum difference in sensor-level slope) that occurred before and after deviant SSSSS blocks; (3) individual source-level P300 amplitude elicited by deviant SSSSS blocks and the strength of inter-block CMV that occurred before and after deviant SSSSS blocks.

## Results

### Magnetic Somatosensory Mismatch Negativity

The number of epochs taken into account for msMMN analyses was 95 ± 8 (mean ± standard deviation (SD) over subjects).

**Figure 2:**
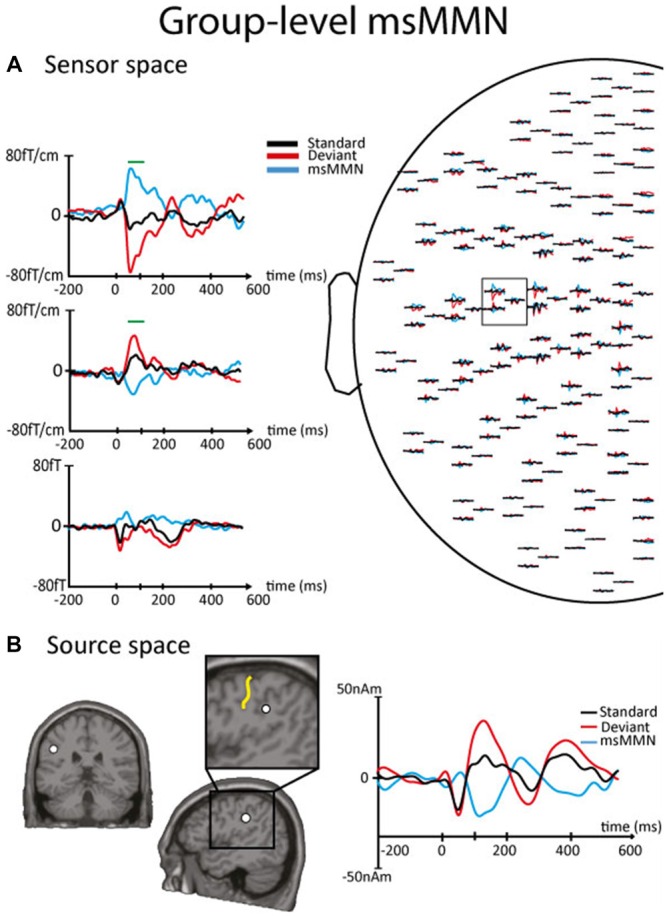
**Results of the msMMN obtained in the sensor and the source spaces at the group level in session 1. (A)** Right. Left part of the magnetoencephalography (MEG) sensor array viewed from top. Left. Enlarged orthogonal planar gradiometers (Top, Middle) and magnetometer (Bottom) signals showing evoked magnetic responses corresponding to standards (black line) and local deviants (red line). The blue line corresponds to the msMMN. Green lines indicate the timing of significant differences between standards and deviants disclosed by non-parametric cluster-based statistics performed at the sensor level. Of notice, in the sensor space, the polarity of the neural responses may appear different from sensor to sensor due to different spatial sensitivity (i.e., lead field) of orthogonal planar gradiometers and magnetometers to right beneath or nearby neural sources. **(B)** Left-Middle. Sagittal and coronal slices of the left hemisphere showing the location of the equivalent current dipole (ECD; white dot) that best explains the magnetic field pattern at the msMMN maximum amplitude. The enlarged area of the sagittal slice shows that the ECD is located at the posterior bank of the left postcentral sulcus, i.e., at the rostroventral part of the left inferior parietal lobule. The central sulcus is indicated by the yellow line. Right. Source waveforms corresponding to standards (black line) and local deviants (red line). The msMMN (blue line) shows a negative deflection.

**Figure 3:**
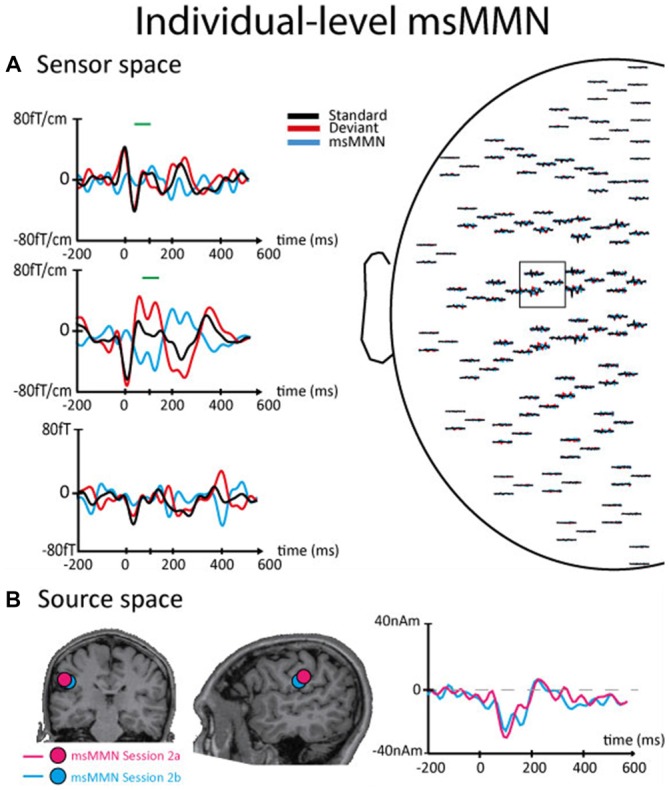
**Results of the msMMN obtained in the sensor and the source spaces for a typical subject in session 2a (standards and deviants similar as in session 1) and 2b (standards and deviants were flipped compared to sessions 1 and 2a). (A)** Results obtained at the sensor level for session 2b. Please, refer to the legend of Figure 2 for more information about this part of the figure. **(B)** Left and Middle. Coronal (left) and sagittal (middle) slices of the brain showing the location of the ECDs that best explains the cortical generator of the msMMN in sessions 2a (pink dot) and 2b (blue dot). Right. Superposition of msMMN waveforms corresponding to sessions 2a (pink line) and 2b (blue line).

Figures 2, 3 illustrate the msMMN results obtained in the sensor and the source spaces (Figure 2, session 1, group level; Figure 3, session 2, individual level).

In session 1, local deviants elicited a significant msMMN peaking at 55–130 ms post-deviant over the left central sensors in 12 out of 16 subjects. The location of the msMMN cortical generator was compatible with the secondary somatosensory (SII) cortex contralateral (cSII) to the stimulation in all subjects. Similarly, at the group level, when concentrating on the time-window of individual-level msMMN occurrence, local deviants elicited significant msMMN peaking at 70–100 ms post-deviant over the posterior bank of the postcentral sulcus, i.e., at the rostroventral part of the left inferior parietal lobule (MNI coordinates, *x*: −47, *y*: −22, *z*: 35).

*Post hoc* analyses revealed that the maximum intensity of the cSII cortex source was significantly higher for deviants than for standards (28.02 ± 13.47 nAm vs. 9.55 ± 7.28 nAm, *p* < 0.001). Likewise, it was significantly higher for deviants occurring after a deviant SSSSS block than for those occurring just before (32.59 ± 11.53 nAm vs. 25.54 ± 9.12 nAm, *p* = 0.013); leading to higher msMMN amplitude (−17.3 ± 13.7 nAm vs. −6.8 ± 8.9, *p* = 0.002).

In session 2, flipping standard and (local) deviant stimuli in session 2b (compared with sessions 1 and 2a) elicited a significant msMMN peaking at 70–180 ms post-deviant over the left central sensors in three out of the five subjects. This msMMN response was similar to those observed in session 2a (Figure 2B). In session 2c where deviant and standard blocks were reversed (compared with sessions 1, 2a and 2b) leading to less frequent and predictable local deviants, a significant msMMN peaking at 75–155 ms post-deviant over the left central sensors was also found in four out of five subjects. There was no significant statistical difference at the sensor level between deviants in session 2a vs. 2b, session 2a vs. 2c nor between standards in sessions 2a, 2b and 2c.

As in session 1, the location of msMMN cortical source in sessions 2 (a, b and c) was compatible with cSII cortex. *Post hoc* analyses showed that maximum cSII source intensity was significantly higher for local deviants occurring in deviant SSSSD blocks (session 2c) than in standard SSSSD blocks (session 2a; 33.7 ± 8.28 nAm vs. 19.46 ± 7.4 nAm, *p* = 0.004); leading to higher msMMN amplitude when local deviants were less predictable (−25.1 ± 5.13 nAm vs. −10.9 ± 4.6, *p* = 0.004).

### P300

Subjects detected 19.26 ± 1.43 deviant SSSSS blocks out of the 20 that were presented in session 1.

**Figure 4:**
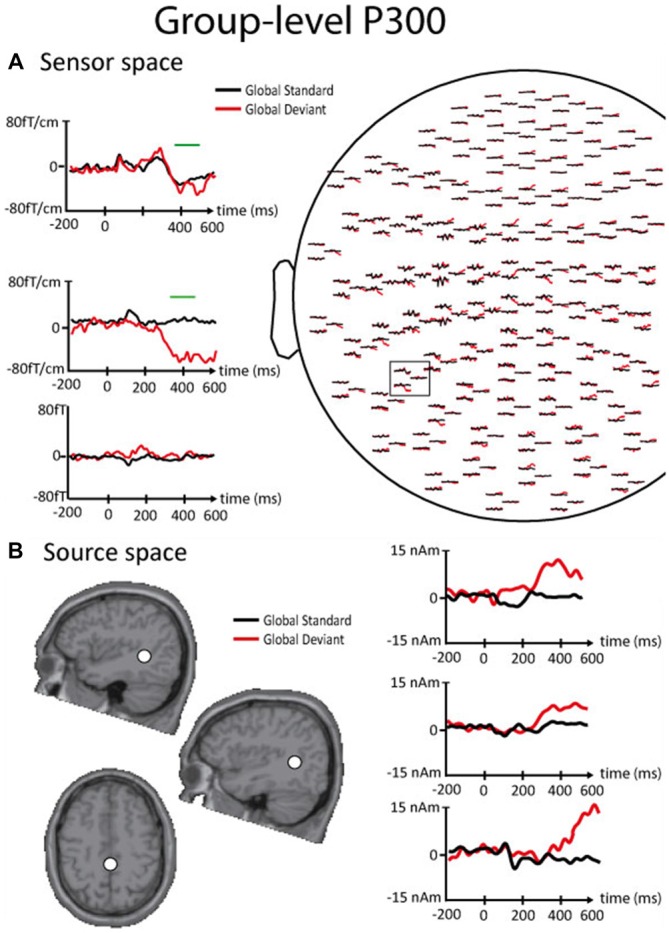
**Results of the P300 response obtained in the sensor and the source spaces at the group level. (A)** Right. Left part of the MEG sensor array viewed from top. Left. Enlarged orthogonal planar gradiometers (Top, Middle) and magnetometer (Bottom) signals showing evoked magnetic responses corresponding to global standards (black line) and global deviants (red line). The green lines indicate the timing of significant differences between standards and deviants disclosed by non-parametric cluster-based statistics performed at the sensor level. **(B)** Right. Source waveforms corresponding to global standards (black line) and global deviants (red line) for the different sources (Top, left temporo-parietal junction (TPJ); Middle, right TPJ, Bottom, supplementary motor area (SMA)). Left. Locations of the ECDs (white dot) that best explain the magnetic field pattern corresponding to the P300 response. Top. Sagittal slice of the left hemisphere. Middle. Sagittal slice of the right hemisphere. Bottom. Axial slice presented in the neurological convention.

Figure 4 illustrates the P300 results obtained at the group level in the sensor and the source spaces in session 1.

The number of epochs taken into account for P300 analyses at the subject-level was 19 ± 1.4 and the P300 grand average was computed with 305 trials obtained from the 16 subjects that remained from the initial 320 trials after artifact rejection. At the group level, global deviants elicited a significant P300 peaking at 340–460 ms post-stimulus over the midline and the temporo-parietal sensors bilaterally. Cortical sources were located at the temporo-parietal junction (TPJ; right TPJ; *x*: 40, *y*: −51, *z*: 12/left TPJ; *x*: −42, *y*: −45, *z*: −9) bilaterally and at the supplementary motor area (SMA; *x*: −4, *y*: 16, *z*: 54). The spatiotemporal patterns of these three cortical sources suggested a sequential activation with TPJ sources being active earlier (~300 ms post-stimulus) than the SMA (~400 ms post-stimulus).

At the individual level, global deviants elicited P300 responses in 14 out of 16 subjects, but that reached statistical significance in only 3 out of 16 subjects.

### Expectation

**Figure 5:**
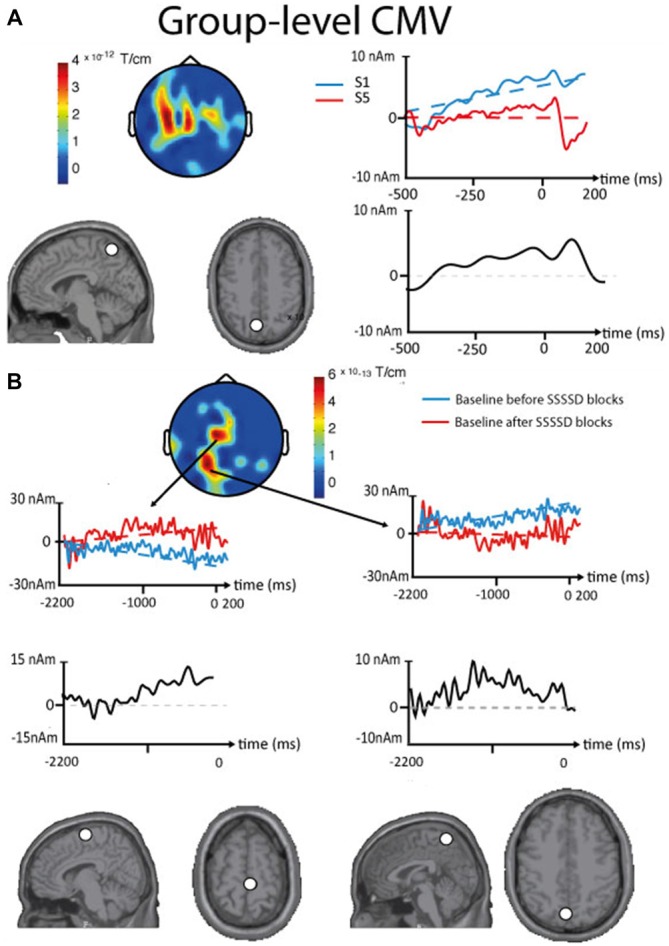
**Results of the contingent magnetic variation (CMV) baseline drift obtained in the sensor and the source spaces at the group level. (A)** Intra-block CMV. Top, left. Topographic plot (sensors array viewed from the top, Euclidian norm of orthogonal planar gradiometers) locating the significant differences in baseline drift between the first (S1) and the fifth stimuli (S5) of standard SSSSD blocks. Top, right. Time courses from one significant sensor with their linear fit (dotted lines). Bottom, left. Axial and sagittal slices presented in the neurological convention showing the location of the ECD (white dot) that best explains the intra-block CMV. Bottom, right. Source waveform corresponding to the intra-block CMV. **(B)** Inter-block CMV. Top, Middle. Topographic plot (sensors array viewed from the top, Euclidian norm of orthogonal planar gradiometers) locating the significant differences in baseline drift between the standard SSSSD blocks occurring before and after deviant SSSSS blocks. Left and Right. Time courses from significant sensors with their linear fit (dotted lines). Middle. Source waveforms corresponding to the intra-block CMV. Bottom. Axial and sagittal slices presented in the neurological convention showing the locations of the ECDs (white dot) that best explain the inter-block CMV baseline drift (Left, SMA; Right, PPC).

Figure 5 illustrates the results of intra- and inter-block CMV analyses obtained in session 1.

#### Intra-Block CMV

In all subjects, a baseline drift was found at the intra-block level (standard SSSSD blocks) between the first somatosensory stimulus and the expected fifth (deviant) stimulus of the sequence. This baseline drift was maximal over midline central sensors with statistical significance over central and left frontal sensors. ECD modeling performed using the grand averaged data identified the posterior parietal cortex (PPC; *x*: −9, *y*: −57, *z*: 60) as the likely cortical generator of the baseline drift.

#### Inter-Block CMV

At the group level, a significant baseline drift was observed between the baseline of standard SSSSD blocks occurring before and after deviant SSSSS blocks. On the grand average, this difference was maximal over right frontal and midline central sensors with statistical significance over central and posterior midline sensors, with the PPC (*x*: −6, *y*: −61, *z*: 55) and the SMA (*x*: 8, *y*: −15, *z*: 66) as likely cortical sources.

### Correlations Between msMMN, P300 and CMV

In session 1, a significant positive correlation between the amplitudes of the msMMN and the P300 was disclosed at the right TPJ (Spearman’s correlation coefficient *R* = 0.58, *p* = 0.02). A significant negative correlation between the difference in msMMN amplitude occurring before and after deviant SSSSS blocks and the concomitant strength of the inter-block baseline drift was also observed (*R* = –0.53; *p* = 0.04). After correction for multiple comparisons, a trend remained between msMMN and P300 (*p* = 0.02 for corrected significance level at 0.017) and between msMMN and CMV (*p* = 0.04 for corrected significance level at 0.017) that failed to reach statistical significance. On the other hand, the correlation was not significant between the CMV and the P300 amplitudes (*R* = –0.24, *p* = 0.39 at right TPJ; *R* = −0.2, *p* = 0.48 at left TPJ; and *R* = 0.27, *p* = 0.33 at SMA).

## Discussion

Using a mechanical unilateral tactile oddball paradigm, this study provides novel empirical evidence favoring a multilevel cortical processing for somatosensory novelty detection that involves multiple segregated cortical regions (cSII cortex, TPJ, SMA and PPC).

At the preattentive level (individual and group-level analyses), tactile novelty elicited a msMMN peaking at 55–130 ms post-local deviants. The timing of this mismatch response, and the location of its neural source, argue for a cortical generator located at putative cSII cortex. This response timeframe indeed do not match with SI cortex response, which typically occurs 30–60 ms post tactile stimulation (Papadelis et al., 2011; Avanzini et al., 2016). At the group level, the msMMN neural source was localized at the rostroventral part of the left inferior parietal lobule; a location not compatible with SI cortex but with a MNI *z* coordinate not typical of SII cortex responses. This discrepancy between the group-level MNI coordinates of the msMMN cortical source and the typical anatomical location of SII cortex might be related to the relatively limited spatial resolution of MEG. Still, these group-level MNI coordinates correspond to the edges (low probability) of the parietal operculum probabilistic maps described by Eickhoff et al. (2006). In session 1, a significant msMMN was found in 75% of the included subjects, which is in agreement with the occurrence of auditory MMN in healthy adult subjects (most of the sMMN data available in the literature being reported at the group level; Kekoni et al., 1997; Shinozaki et al., 1998; Akatsuka et al., 2005; Restuccia et al., 2007; Spackman et al., 2007; Butler et al., 2011; Strömmer et al., 2014). Furthermore, although performed on a limited number of subjects, the comparison of msMMN results obtained in session 2a and 2b suggested that msMMN responses observed in session 1 were not only related to the change in the physical characteristics of standard and deviant tactile stimulations. The spatiotemporal dynamics of msMMN observed in this study is in agreement with some previous EEG and MEG studies that used somatosensory oddball paradigms (Shinozaki et al., 1998; Akatsuka et al., 2005; Restuccia et al., 2007; Butler et al., 2011; Strömmer et al., 2014). Indeed, the present data and most of the previous studies disclosed that sMMN involves central regions at 100–200 ms post-deviant with likely neural generator located at the SII cortex (Akatsuka et al., 2005).

SII cortex is known to play a key role in selective attention upon elaborated stimuli characteristics such as timing, space location, pain, laterality or their surprising aspect (Fujiwara et al., 2002; Hamäläinen et al., 2002; Simões et al., 2002; Zhu et al., 2007; Chen et al., 2008, 2010; Ostwald et al., 2012). These integrative features of SII cortex and the results of sMMN studies highly suggest that the SII cortex in humans plays a pivotal role for the early (preattentional) stage of somatosensory novelty detection.

In the auditory modality, different theories are opposed to account for the mechanisms of MMN. In the *adaptation* theory, MMN represents a subtraction artifact due to attenuation and delay of N100 response to an auditory stimulus as a function of its similarity to the preceding auditory events, reflecting short-lived adaptation of auditory cortex neurons (Jääskeläinen et al., 2004; Garrido et al., 2007a). A second theory deems that the MMN reflects a *model adjustment* due to the comparison of the auditory input with the memory trace of previous sounds (Garrido et al., 2007a; Näätänen et al., 2011). Finally, the *predictive coding* theory unifies the adaptation and the adjustment hypotheses by considering that specific neuronal error prediction units generate MMN when a novel incoming stimulus do not match with the predictions about incoming stimuli (adjustment) and that the prediction error is lessened when there is a high variance in incoming stimuli (adaptation; for a review, see Garrido et al., 2009b). This implies a top-down influence on mismatch responses with higher MMN amplitude when deviants are less predictable.

Results of the present study argue in favor of the predictive coding theory for the mechanism involved in sMMN. First, our observation that msMMN amplitude is increased when the occurrence of the local deviant is less predictable (i.e., when a deviant block breaks the prediction pattern set by the repetition of previous standard blocks or when local deviants occur in deviants blocks that are less frequent and randomly inserted) suggests a top-down influence on low-level, preattentive cortical responses. Second, indirect arguments for the predictive coding theory come from the finding that the msMMN in the somatosensory modality is mainly generated at SII cortex. Indeed, if somatosensory adaptation phenomena have been demonstrated in SI cortex, SII cortices and PPC using electrical median nerve stimulation (Wikstrom et al., 1996; Mauguière et al., 1997), adaptation to ecological tactile stimuli similar to those used in this study seems to happen essentially at the SI cortex and PPC (Popescu et al., 2013). Interestingly, in the latter study, massive suppression occurred at SII cortex after the first stimulus in a sequence of identical stimuli (Popescu et al., 2013). This finding could also be interpreted in the context of the predictive coding framework, i.e., repeated stimuli did not lead to SII cortex activation because they matched the prediction. Further arguments in favor of the predictive coding theory as the likely mechanism involved in the auditory MMN came from auditory omission paradigms, i.e., oddball paradigms in which deviants consisted in omitted stimuli. Indeed, in those studies, omissions also elicited mismatch responses (Wacongne et al., 2011) that could be explained as a response to prediction error but not by the adaptation theory. In addition, responses to omitted auditory stimuli appeared to be modulated by expectation (Todorovic et al., 2011; Todorovic and de Lange, 2012), therefore favoring the predictive coding over the adjustment theory. Interestingly, in the somatosensory modality, such “omission responses” have also been recorded at SII cortex when recurrent somatosensory stimuli abruptly stopped (Yamashiro et al., 2008). Even if the mechanisms involved in the msMMN should be properly addressed by further dedicated MEG/EEG studies, these data suggest that the msMMN is likely to be generated at the SII cortex under the predictive coding framework.

At the attentive level (group-level analysis), we found a component with a positive polarity starting around 300 ms post stimulus that involved a sequential activation of bilateral TPJs and SMA. Such distribution and timing features are highly suggestive of the P300 response described to global deviants in the auditory version of the local/global paradigm (Bekinschtein et al., 2009; Wacongne et al., 2011; Chennu et al., 2013). Interestingly, in similar auditory oddball paradigms, P300 responses were reported whether subjects counted the number of deviant blocks (Chennu et al., 2013) or were merely instructed to pay attention to the stimuli (Wacongne et al., 2011). In this study, subjects were asked to count the number of deviant SSSSS blocks to insure that attention between subjects was equivalent (as indexed by a similar rate of deviant block detection (>90%)) to support group-level analyses. Initially, the P300 response was considered to reflect working memory update (Polich, 2007). The development of the integrative auditory *local/global* oddball paradigm (Bekinschtein et al., 2009; Wacongne et al., 2011; Chennu et al., 2013) demonstrated that P300 responses are also related to the conscious detection of complex changes in a continuous stream of sensory inputs by a high-order novelty-sensitive system (Wacongne et al., 2011). P300 responses are indeed considered to index conscious access to identified patterns of sensory inputs and their deviations (Dehaene and Changeux, 2011), leading to their use for the detection of conscious sensory processing in patients with disorders of consciousness (Bekinschtein et al., 2009; Faugeras et al., 2012; Sitt et al., 2014). At the individual level, we found this response in a lower proportion of subjects than previously reported in the auditory studies using a similar paradigm. This is explained by the fact that in those studies, P300 responses were identified subject-wise across multiple sessions with global deviants (leading to over 100 trials averaged for each subject), while we had at most 20 global deviant trials for each subject. Nonetheless, our grand average data identified the TPJs as neural generators of the P300, with locations similar to those previously disclosed for the auditory modality (Bekinschtein et al., 2009; Wacongne et al., 2011; Chennu et al., 2013). The coordinates of our TPJ sources were also consistent with fMRI data showing TPJ involvement in the detection of salient stimuli across multiple modalities (Downar et al., 2000; Blanke, 2012). TPJ, mostly in the right hemisphere, is also a cornerstone of the ventral attention network responsible for the detection of unexpected but behaviorally relevant events (Corbetta and Shulman, 2011). The trend for a correlation between the amplitude of msMMN generated at the putative SII cortex and the amplitude of the P300 generated at the right TPJ is therefore of high interest as it might suggest the existence of a hierarchical framework for tactile change detection. Dedicated studies using effective connectivity methods such as dynamic causal modeling (garrido et al., 2007b) should therefore be done to confirm this hypothesis.

The subsequent involvement of the SMA in P300 generation and its location are in line with previous fMRI and MEG studies that used somatosensory or multimodal oddball paradigms (Downar et al., 2000; Huang et al., 2005). The observed SMA activity could be interpreted as reflecting the planning of a covert motor response triggered by novel and behaviorally relevant somatosensory input (Downar et al., 2000). Alternatively, the SMA might be involved as such in the neural network responsible for the detection of salient sensory event, as suggested by clinical observations in patients suffering from neglect due to SMA lesions (Vallar, 1998).

Finally, CNV is thought to reflect progressive changes in expectation based on the hypothesis that expectations about incoming stimuli are supposed to increase with each correct prediction of sensory inputs. CNV was initially described in the “S1-S2 paradigm” where a warning signal (S1) was followed by a target stimulus several seconds later (S2) towards which subjects were instructed to act; CNV being observed in the lapse between S1 and S2. The neural generator of CNV was found at the SMA and CNV was consequently initially explained by *motor anticipation*, i.e., the preparation of an action induces a concurrent expectation of when that action is likely to be executed (for a review, see Mento et al., 2013). Still, there are increasing evidence for CNV-like responses in paradigms that do not engage any action-related mechanism. Indeed, a baseline drift similar to CNV was found in both auditory (Faugeras et al., 2012; Chennu et al., 2013) and visual (Mento et al., 2013) oddball paradigms after successive repetitions of the standard stimuli combination that lead to progressive inference or expectancy about environmental regularity. We found a similar baseline drift after successive repetitions of expected somatosensory stimuli or sequences that was maximal over midline sensors. This baseline drift is highly suggestive of a somatosensory magnetic counterpart of the CNV elicited by the integrative auditory local/global paradigm. Indeed, stimulation paradigms are equivalent and the neural sources (PPC, SMA) explaining the observed drift concur with those previously described for CNV in EEG (Gómez et al., 2004; Faugeras et al., 2012; Mento et al., 2013, fMRI) and CMV in MEG (Hultin et al., 1996; Dammers and Ioannides, 2000).

In summary, this study demonstrates the existence of a multilevel cortical processing of somatosensory novelty detection. Specifics of the somatosensory cortical network functional segregation (by comparison with, e.g., the auditory cortical network where primary and secondary areas are very close to each other anatomically) allowed us to locate the preattentional response (MMN) at putative SII cortex, while higher-levels of cortical processing were located at the TPJ, the PPC and the SMA. These findings suggest that the cortical processing of novel sensory stimulation occurring in the personal space shares common features with those occurring in the extrapersonal space, implying a common framework of multilevel cortical processing for sensory novelty in the human brain.

## Auhtor Contributions

GN designed the study, led the experiments, analyzed and interpreted the results and is the main author of the article. TV participated in all experiments and helped analyzing the results. VW and BM wrote Matlabscripts, participated in the experiments and in the analysis and interpretation of the results as well as in the statistics. SG contributed to the study design and to the content and writing of the article. XDT contributed to the design of the study, the analysis and interpretation of the data and the writing of the article.

## Funding

GN is supported by a research grant from the Fonds Erasme (http://www.fondserasme.org/, Brussels, Belgium). VW (Research logistic collaborator) and XDT (Post-doctorate Clinical Master Specialist) are supported by a research grant from the Fonds de la Recherche Scientifique (F.R.S.-FNRS, Belgium). This work is supported by a research grant from the Fondation ULB to Pr SG (Université libre de Bruxelles, Belgium). The MEG project at the ULB-Hôpital Erasme is supported by the Fonds Erasme (http://www.fondserasme.org/, Brussels, Belgium).

## Conflict of Interest Statement

The authors declare that the research was conducted in the absence of any commercial or financial relationships that could be construed as a potential conflict of interest.

## Footnotes

^1^http://fieldtrip.fcdonders.nl/
